# What Did We Accomplish in Fighting Radical Species in Human Health?

**DOI:** 10.3390/antiox10030466

**Published:** 2021-03-16

**Authors:** Rachid Skouta

**Affiliations:** Department of Biology, University of Massachusetts, Amherst, MA 01003-9297, USA; rskouta@umass.edu; Tel.: +1-413-577-4167

Maintaining the physiological level of reactive oxygen species (ROS) and reactive nitrogen species (RNS) in the body is highly important in the fight against radical species in the context of human health. An excess of ROS and RNS, also called the bad radical species, plays a critical role in many diseases, including cancer and neurodegeneration. Therefore, the development of new approaches based on small molecules mitigating the above physiological level of ROS/RNS is crucial. For example, identifying new therapeutic strategies capable of modifying the course of neurodegenerative diseases such as amyotrophic lateral sclerosis (ALS), Parkinson’s disease, autism, and migraine, is currently one of the major goals of researchers in this field. Strong evidence suggests that natural and non-natural antioxidant compounds are alternative therapeutics that can be used to mitigate oxidative stress in various models [1,2]. Natural antioxidant plant-based compounds, such as curcumin (e.g., *Curcuma longa*) and resveratrol (e.g., cranberry—Vaccinium macrocarpon), are highly abundant and accessible with low toxicity and side effects. On the other hand, non-natural antioxidants are a class of compounds created from a well-defined synthetic route that are highly efficacious but less accessible and may have considerable toxicity (e.g., ferrostatin-1, a lipophilic antioxidant inhibitor of ferroptosis cell death) [3,4]. Therefore, further investigations of additional resources of natural antioxidants are needed to reduce oxidative stress in the body safely.

In this Special Issue entitled “Fighting Radical Species in Human Health: Mitigating Radical Species with Natural and Synthetic Compounds”, we discuss a broad range of multidisciplinary investigative contributions, spanning from neuroscience, cancer, chemical biology, and plant biology to nutraceuticals with antioxidative properties. In an elegant review of this issue, Remigante et al. [5] provided an insightful discussion on the mechanism of action of band 3 protein (B3p) in erythrocyte, a simple in vitro model to study the impact of antioxidants on cellular homeostasis. B3p protein is responsible for exchanging Cl^−^ and HCO_3_^−^ anions through erythrocyte membranes and for acid balance, ion distribution, and gas exchange, thus accounting for homeostasis of both erythrocytes and entire organisms. The review also highlights the benefit of natural antioxidants, such as curcumin, magnesium, and melatonin, in blocking B3p anion exchange capability under in vitro oxidative conditions, suggesting that antioxidant supplements may be useful in improving endogenous antioxidants and increase the body defense machinery. Morán-Santibañez et al. [6] reported in an original paper the potential antioxidant properties of Creosote bush (Larrea tridentata, LT) leaves (collected from the Chihuahuan desert in the region of El Paso del Norte (TX, USA) [7]. LT leaf extracts were tested for their potential efficacy to mitigate cellular oxidative stress on human neuroblastoma SH-SY5Y cells as an in vitro model of PD. Overall, the LT extract exhibited a protective effect on SH-SY5Y cells undergoing oxidative stress in vitro, functioning as a natural anti-apoptotic extract.

On the other hand, a review by De Lazzari et al. [8] discussed the clinical relevance of the use of *Drosophila melanogaster* (fruit flies) as an animal model to evaluate the therapeutic potential of natural and synthetic antioxidants therapy in PD.

Protein aggregation is primarily characterized by the degenerative human ALS disease. ALS is a common degenerative disease of the central nervous system concerning a progressive loss of upper and lower motor neurons. As the wobbler mouse and ALS show striking similarities in view of phenotypical attributes, the wobbler mouse is rated as an animal model for the disease. In an original paper, Zwilling et al. [9] reported that nicotinamide adenine dinucleotide (NAD^+^) acts as a neuroprotector which facilitates the reduction in oxidative stress. NAD^+^ is primarily known as an essential co-enzyme in energy metabolism, gene expression, and DNA repair. The authors investigated the effect of NAD^+^ enhancement on motor neuronal development in an in vitro model of the wobbler mouse. They also reported that the small molecule caffein, a known antioxidant compound, increased the level of nicotinic acid mononucleotide transferase 2 (Nmnat2) specifically in neuronal cells. The authors concluded that caffeine as well as NAD^+^ were both shown to have a positive impact on the in vitro development of motor neurons of wobbler mice. This study represents a significant advance toward uncovering potential pharmacological treatments of both wobbler and ALS disease.

Polyphenolic natural products are known to exhibit significant anti-oxidative and anti-inflammatory properties. Altered immune alteration and oxidative stress have also been found in patients with autism spectrum disorders (ASD), and these alterations could add to the pathophysiology associated with ASD. The review conducted by Malaguarnera et al. [10] focused on the benefit of resveratrol in ASD. The stilbene resveratrol is a polyphenolic small molecule with substantial anti-oxidative and anti-inflammatory properties recently tested in animal models of several neurological diseases.

Headache disorders including migraine are still underdiagnosed and lack efficacious treatments. Therefore, the development of a new classes of treatments is desirable. Goschorska et al. [11] elucidated the use of antioxidants such as vitamin C, curcumin, coenzyme Q_10_, and ginkgolide B in the treatment of migraine. The present review provided a summary of the studies on nutraceuticals with antioxidative properties. The results presented therein seem to indicate the possible use of nutraceuticals with antioxidative properties as an alternative to conventionally used medication in migraine treatment.

Natural-based compounds bearing antioxidants and anti-inflammatory properties are highly useful in mitigating oxidative stress. Oxidative stress and inflammation are reported to be among the major contributors to cancer. Considering the key role of oxidative stress during acute pancreatitis (AP), Cordaro et al. [12] tested the effect of one of the main sources of polyphenols in the diet worldwide, cashew nuts (*Anacardium occidentale* L. originated from Ivory Coast, Africa), on pancreatic and lung injury induced by cerulein injection in vivo. In this study, cashew nuts showed many benefits in several cellular pathways. For example, the levels of nucleotide-binding domain leucine-rich repeat containing family pyrin domain containing receptor 3 (NLRP3), the apoptosis-associated speck like protein containing a caspase recruitment domain (ASC), and caspase-1 were significantly increased after cerulein induction, and cashew nuts considerably diminished this increase in both the pancreas and lung. Additionally, the activation of the nuclear factor E2-related factor 2 (Nrf2) pathway was boosted, which suggests its antioxidant properties. In another original manuscript, Fusco et al. [13] reported the use of hydroxytyrosol, a known phenolic small molecule obtained from olive oil, in a pancreatitis-associated gut injury in vivo model. Specifically, the pharmacological effect of hydroxytyrosol treatment prior to cerulein (a pancreatic and intestinal injury inducer) administration on a CD1 female mice was efficacious in decreasing amylase and lipase (the serum hallmarks of pancreatitis). In addition, the lipid peroxidation and oxidative stress levels (e.g., superoxide dismutase and glutathione peroxidase) decreased, suggesting that hydroxytyrosol may be an important therapeutic tool against pancreatitis-induced injuries in the pancreas and gut.

Park et al. [14] examined in an original manuscript the mechanism of action of the known dietary antioxidant β-carotene in gastric epithelial cells infected with Helicobacter pylori. The pretreatment of cells with β-carotene prior to helicobacter pylori infection significantly attenuates the overall oxidative stress (e.g., nicotinamide adenine dinucleotide phosphate (NADPH) oxidase and nuclear factor kappa-light-chain-enhancer of activated B (NF-kB)), suggesting that dietary supplementation with β-carotene-rich foods may prevent or delay the development of gastric diseases, including gastric cancer, associated with Helicobacter pylori infection.

Finally, identifying novel natural compounds and nutraceuticals bearing antioxidant properties will significantly enhance our understanding of their roles against the bad radical species in biological systems. Eventually, this may lead to the design of novel mono- or combined therapeutic strategies offering more effective treatments that will positively impact clinical outcomes and human health.
